# HIV‐1 transmitted drug resistance surveillance: shifting trends in study design and prevalence estimates

**DOI:** 10.1002/jia2.25611

**Published:** 2020-09-16

**Authors:** Soo‐Yon Rhee, Seble G Kassaye, Geoffrey Barrow, Jagadish Chandrabose Sundaramurthi, Michael R Jordan, Robert W Shafer

**Affiliations:** ^1^ Department of Medicine Stanford University Stanford CA USA; ^2^ Department of Medicine Georgetown University Washington DC USA; ^3^ Department of Medicine Faculty of Medical Science University of the West Indies Mona Jamaica; ^4^ Division of Geographic Medicine Tufts Medical Center Boston MA USA; ^5^ Department of Public Health and Community Medicine Tufts University School of Medicine Boston MA USA; ^6^ Tufts Center for Integrated Management of Antimicrobial Resistance (CIMAR) Boston MA USA

**Keywords:** HIV‐1, antiretroviral therapy, drug resistance, surveillance, reverse transcriptase, protease

## Abstract

**Introduction:**

HIV‐1 transmitted drug resistance (TDR) prevalence increased during the initial years of the antiretroviral therapy (ART) global scale‐up. Few studies have examined recent trends in TDR prevalence using published genetic sequences and described the characteristics of ART‐naïve persons from whom these published sequences have been obtained.

**Methods:**

We identified 125 studies published between 2014 and 2019 for which HIV‐1 reverse transcriptase (RT) with or without protease from ≥50 ART‐naïve adult persons were submitted to the GenBank sequence database. The population characteristics and TDR prevalence were compared to those in 122 studies published in the preceding five years between 2009 and 2013. TDR prevalence was analysed using median study‐level and person‐level data.

**Results and discussion:**

The 2009 to 2013 and 2014 to 2019 studies reported sequence data from 32,866 and 41,724 ART‐naïve persons respectively. Studies from the low‐ and middle‐income country (LMIC) regions in sub‐Saharan Africa, South/Southeast Asia and Latin America/Caribbean accounted for approximately two‐thirds of the studies during each period. Between the two periods, the proportion of studies from sub‐Saharan Africa and from South/Southeast Asia countries other than China decreased from 43% to 32% and the proportion of studies performed at sentinel sites for recent HIV‐1 infection decreased from 33% to 22%. Between 2014 and 2019, median study‐level TDR prevalence was 4.1% in South/Southeast Asia, 6.0% in sub‐Saharan Africa, 9.1% in Latin America/Caribbean, 8.5% in Europe and 14.2% in North America. In the person‐level analysis, there was an increase in overall, NNRTI and two‐class NRTI/NNRTI resistance in sub‐Saharan Africa; an increase in NNRTI resistance in Latin America/Caribbean, and an increase in overall, NNRTI and PI resistance in North America.

**Conclusions:**

Overall, NNRTI and dual NRTI/NNRTI‐associated TDR prevalence was significantly higher in sub‐Saharan Africa studies published between 2014 and 2019 compared with those published between 2009 and 2013. The decreasing proportion of studies from the hardest hit LMIC regions and the shift away from sentinel sites for recent infection suggests that global TDR surveillance efforts and publication of findings require renewed emphasis.

## 1 Introduction

HIV‐1 drug resistance (HIVDR) testing is not routinely available for clinical management in most low‐ and middle‐income countries (LMICs), which shoulder the largest global burden of HIV‐1 infection. The choice of initial antiretroviral therapy (ART) in LMICs is thus informed by population‐level HIVDR prevalence estimates derived from persons initiating therapy. WHO initially recommended methods to classify the prevalence of transmitted drug resistance (TDR) in persons likely to have been recently infected with HIV. Such surveys were designed to maximize inclusion of virus with drug resistance mutations (DRM) that had been transmitted before they were outcompeted by more fit wild‐type revertants [1, 2]. Such surveys, however, were not nationally representative and in low incidence settings, restrictive participant inclusion criteria made them challenging to complete enrolment in a reasonable time period [3].

In 2015, the WHO modified its HIV drug resistance surveillance strategy to focus primarily on populations initiating first‐line ART; resistance detected in these surveys was called pretreatment drug resistance (PDR) [4]. In contrast to studies in which all persons were antiretroviral (ARV) drug‐naïve, PDR surveys allowed for the inclusion of persons initiating (or re‐initiating) first‐line ART who may have previously received ARV drugs such as those who received ARVs for the prevention of mother‐to‐child transmission or in whom prior ART was interrupted. This surveillance strategy assesses the overall burden of HIVDR in the population initiating and reinitiating ART and best supports ART guideline optimization within the public health approach in most LMICs.

WHO spearheaded two systematic reviews of HIVDR in previously ART‐naïve persons from LMICs. The first published in 2012, reported an increase in TDR in southern and eastern Africa between 2001 and 2011 [5]. The second review published in 2018, examined PDR studies through 2016 and reported that in multiple regions in sub‐Saharan Africa and in Latin America/Caribbean region, non‐nucleoside reverse transcriptase inhibitor (NNRTI) PDR levels had approached or exceeded 10% [6]. Similar high levels of NNRTI‐associated TDR and/or PDR have been reported in systematic reviews from South Africa [7] and the Latin America/Caribbean region [8, 9].

In 2015, we published a systematic review and meta‐analysis of global TDR trends using data from 287 studies published between 2000 and 2013. Our 2015 meta‐analysis differed from previous reviews and meta‐analyses because it included only studies for which HIV‐1 sequences had been submitted to GenBank [10]. The availability of all sequences made it possible to perform individual person‐level analyses and to analyse trends in the prevalence of each drug resistance mutation. The study reported a yearly 1.09‐fold increase in the odds of TDR since global ART scale‐up programmes began and reported that NNRTI resistance increased in five regions including sub‐Saharan Africa, Latin America/Caribbean, North America, Europe, as well as in upper‐income Asian countries.

In this study, we describe TDR prevalence data collected in the six years since the completion of our previous meta‐analysis (2014–2019). We compare the population characteristics and prevalence of TDR in recent studies to those studies published in the preceding five‐year period between 2009 and 2013 meeting the same inclusion criteria. Like our previous study, the current analysis includes only those studies for which sequences are publicly available.

## 2 Methods

### 2.1 Study inclusion criteria

All published HIV‐1 group M pol nucleic acid sequences containing the reverse transcriptase (RT) gene of HIV‐1 with or without the protease gene submitted between 1 January 2014 and 15 December 2019 were retrieved using a BLAST search of the GenBank viral sequence database V. 235 (released 15 December 2019) (Table S1). Sequences with the same GenBank “Author” and “Title” fields were grouped into submission sets. The GenBank annotation and associated published papers were reviewed to identify submission sets (studies) describing ≥50 ART‐naïve infected individuals containing sequences encompassing RT codons 40 to 240. We compared the data from these studies with the data from those published between 2009 and 2013 meeting the same criteria.

Studies of individuals initiating first‐line ART were excluded if they also included individuals with any previous ART exposure. Studies that included children born to mothers receiving ARTs to prevent mother‐to‐child transmission were excluded as were studies of populations whose viruses were sequenced based on knowledge of their HIV drug resistance status (i.e. pretherapy sequences from persons who subsequently developed virological failure and/or HIVDR). Next‐generation sequencing (NGS) studies were excluded if the threshold for mutation detection was <15% or not reported.

### 2.2 Data collection

For each study, the following information was collected: (i) Country and year of sampling; (ii) Location at which samples for genotypic resistance testing were obtained including HIV clinics, voluntary counselling and testing centres, antenatal clinics, blood transfusion centres, sexually transmitted diseases clinics and clinics for persons who inject drugs. For some studies, however, information was provided only on the location at which samples underwent genotypic resistance testing (e.g. a reference or public health laboratory). Sentinel sites for HIV‐1 surveillance were defined as sites other than HIV clinics at which genotypic resistance testing was performed; (iii) Stated purpose of the study, such as whether it was performed to estimate TDR prevalence, assess sequence diversity or characterize a transmission network; (iv) Whether the population predominantly comprised recently infected persons; (v) Specimens submitted for sequencing such as plasma, peripheral blood mononuclear cells (PBMCs) and dried blood spots; (vi) Sequencing method used: Sanger sequencing versus NGS. For NGS, the mutation detection threshold was recorded.

Studies meeting inclusion criteria were assigned to one of the following geo‐economic regions: (i) sub‐Saharan Africa; (ii) LMICs of South and Southeast Asia; (iii) Latin America and Caribbean; (iv) Europe; (v) United States, Canada and Puerto Rico (North America); (vi) Upper‐income Asian countries; (vii) Countries of the former Soviet Union; (viii) North Africa (Middle‐east) and (ix) Australia. For studies conducted in countries in different regions, separate datasets for each region were created, provided the study had more than 50 individuals per region. LMICs in sub‐Saharan Africa, South/Southeast Asia and Latin America/Caribbean were defined according to WHO [4].

### 2.3 Sequence analyses

TDR was defined as the presence of one or more mutations from the WHO 2009 list of surveillance drug resistance mutations (SDRMs) [11]. The SDRM list consists of 93 drug resistance mutations including 34 NRTI resistance mutations at 15 RT positions, 19 NNRTI resistance mutations at 10 RT positions and 40 PI resistance mutations at 18 protease positions.

The Calibrated Population Resistance (CPR) analysis tool (https://hivdb.stanford.edu/cpr/) was used to calculate the proportions of individuals per study with overall NRTI, NNRTI and PI‐associated TDR [12]. HIV‐1 subtype was determined using the COMET HIV‐1 Subtyping Tool [13]. For each study, the epidemiologic characteristics, TDR prevalence and CPR analysis can be accessed at http://hivdb.stanford.edu/surveillance/map/.

Tenofovir resistance was defined as the presence of any one of the following four sets of mutations: (i) K65R, K70E and Y115F; (ii) the thymidine‐analogue mutations (TAMs) T215Y/F, which in contrast to the remaining TAMs reduce response to tenofovir‐containing regimens [14]; (iii) the multidrug resistance mutations T69S_SS and Q151M and (iv) several additional non‐polymorphic DRMs not on the SDRM list including A62V, K65N, T69del and K70G/Q/N/S/T [15].

### 2.4 TDR prevalence

TDR prevalence was examined using study‐level analyses in which the median overall and ART‐class TDR prevalences were represented by summary values from each study and individual‐level analyses in which individuals from all studies were pooled. Study‐level analyses compared TDR prevalence in studies submitted to GenBank between 2014 and 2019 and those submitted to GenBank between 2009 and 2013 that contained sequences from ≥50 ARV‐naïve adults. The median prevalence of overall, NRTI, NNRTI and/or PI TDR was compared within those regions for which five or more studies were available in both periods using the Wilcoxon Rank Sum test.

Individual‐level analyses were also performed to examine temporal trends in pooled virus sequences from each region obtained since 2009. This analysis excluded sequences obtained prior to 2009, which represented a substantial proportion of sequences described in the 2009 to 2013 studies and a small proportion of sequences described in the 2014 to 2019 studies. To examine temporal trends since 2009, the overall population of pooled sequences by sample year was bisected and TDR prevalence between the two time periods (2009 to 2011 and 2012 to 2018) was compared using the Fisher Exact test. In a complementary analysis, a generalized linear mixed logistic regression analysis was used to relate the sample year to TDR prevalence. To account for study heterogeneity, study was included in the model as a random effect using the R package lme4.

## 3 Results

### 3.1 Study population

**Figure 1:**
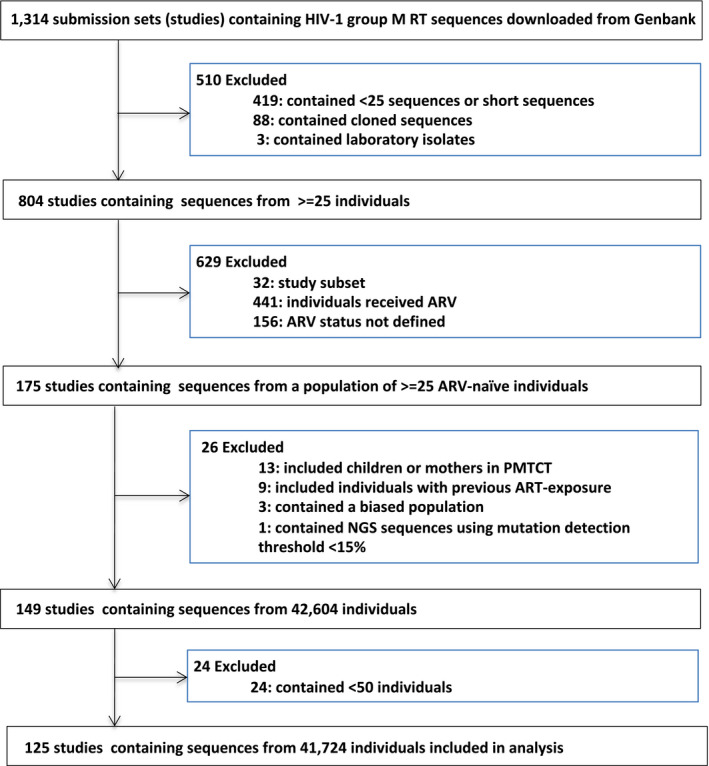
Flow chart showing the derivation of study sets meeting meta‐analysis inclusion criteria.

The 2014 to 2019 GenBank search identified 125 studies meeting study inclusion criteria. Figure 1 displays a flow chart summarizing the process by which studies were reviewed to determine whether they met inclusion criteria. All but four studies were linked to a peer‐reviewed publication. These studies contained RT sequences from 41,724 individuals; a subset of 116 studies contained protease sequences from 40,084 individuals. The list of 125 included studies is provided in Table S2. The comparison dataset included 122 studies submitted to GenBank between January 2009 and December 2013 meeting the same inclusion criteria. The 2009 to 2013 studies contained RT sequences from 32,866 individuals; a subset of 118 studies contained protease sequences from 30,564 individuals.

**Figure 2:**
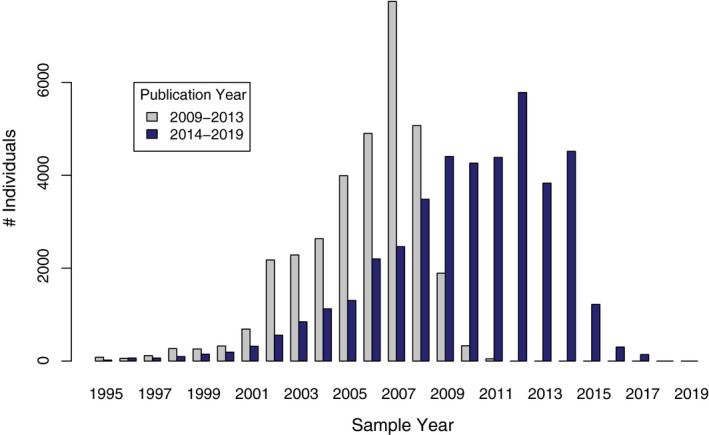
Distribution of sample collection years for studies published between 2009 and 2013 and between 2014 and 2019.

Table 1 summarizes the 125 studies from the 2014 to 2019 period and the 122 studies from the 2009 to 2013 period by geographical region, number of individuals, median sample year, number of countries and most common subtypes. The median number of individuals per study during the 2014 to 2019 period was 145 (IQR: 94 to 290), and the median sample year was 2011 (IQR: 2009 to 2013). The median number of individuals per study during the 2009 to 2013 period was 121 (IQR: 79 to 219), and the median sample year was 2007 (IQR: 2005 to 2008). Figure 2 shows the distribution of sample years for sequences published in GenBank during the 2009 to 2013 and 2014 to 2019 periods. Overall, 39,663 persons had sequences obtained since 2009 including approximately one‐half between 2009 and 2011 and one‐half between 2012 and 2018. The median interval between sample date and publication data was 4 years (IQR: 3 to 5 years).

**Table 1:** Genotypic resistance data in ART‐naïve persons: studies containing ≥50 persons published 2009 to 2013 and 2014 to 2019

	2009 to 2013^a^ (n = 122)	2014 to 2019^b^ (n = 125)
Sub‐Saharan Africa		
Number of studies	35	27
Median individuals per study (IQR)	127 (81 to 188)	96 (78 to 247)
Median Sample year	2008	2012
Number of countries	25	14
Most common subtypes (%)	C (45), A (21), 02_AG (14)	C (73), A (13), 02_AG (7)
South/Southeast Asia		
Number of studies	29	44
Median individuals per study (IQR)	101 (92 155)	188 (123 to 301)
Median Sample year	2009	2012
Number of countries	8	9
Most common subtypes (%)	01_AE (67), B (13), C (8)	01_AE (50), 07_BC (20), B (13)
Latin America/Caribbean		
Number of studies	16	18
Median individuals per study (IQR)	87 (67 to 135)	128 (95 to 198)
Median Sample year	2008	2014
Number of countries	9	6
Most common subtypes (%)	B (87), C (6), F (3)	B (77), C (9), F (9)
Europe		
Number of studies	17	21
Median individuals per study (IQR)	145 (92 to 462)	138 (83 to 341)
Median Sample year	2006	2010
Number of countries	28	15
Most common subtypes (%)	B (66), C (8), 02_AG (7)	B (75), A (6), 02_AG (4)
North America		
Number of studies	13	5
Median individuals per study (IQR)	340 (203 to 662)	645 (345 to 683)
Median Sample year	2005	
Number of countries	3	3
Most common subtypes (%)	B (97), C (1)	B (96), 01_AE (1), C (1)
Upper‐income Asia		
Number of studies	5	5
Median individuals per study (IQR)	133 (76 to 378)	161 (131 to 2129)
Median Sample year	2007	2012
Number of countries	5	3
Most common subtypes (%)	B (87), 01_AE (7), 07_BC (3)	B (88), 01_AE (6), 07_BC (3)
Former Soviet Union Countries		
Number of studies	4	2
Median individuals per study (IQR)	131 (102 to 170)	65 (64 to 67)
Median Sample year	2008	2015
Number of countries	4	1
Most common subtypes (%)	06_cpx (74), A (20), B (5)	A (81), B (13), G (3)
Middle East		
Number of studies	1	3
Median individuals per study (IQR)	80	78 (65 to 307)
Median Sample year	2008	2013
Number of countries	1	3
Most common subtypes (%)	B (76), 02_AG (11), A (5)	B (75), 35_AD (8), A (7)

^a^ 122 studies containing sequences from ≥50 ART‐naïve individuals that were submitted to GenBank in 2009 to 2013; Overall, the number of individuals was 32,866 and the median sample year was 2007 (IQR: 2005 to 2008); 2 studies from Australia containing 627 individuals are not shown

^b^ 125 studies containing sequences from ≥50 ART‐naïve individuals that were submitted to GenBank in 2004 to 2019; Overall, the number of individuals was 41,724 and the median sample year was 2011 (IQR: 2009 to 2013).

Studies from the low‐ and middle‐income regions of sub‐Saharan Africa, South/Southeast Asia and Latin America/Caribbean accounted for 66% of studies during the 2009 to 2013 period and 70% of studies during the 2014 to 2019 period. Between the two study periods, the proportion of studies in sub‐Saharan Africa decreased from 29% to 22% (Fisher Exact test; *p* = 0.2), whereas the number in South/Southeast Asia increased from 24% to 35% (Fisher Exact test; *p* = 0.05). During the first study period, 41% of the 29 studies in South/Southeast Asia were performed in China. In the second study period, 71% of the 44 studies in South/Southeast Asia were performed in China. Therefore, the proportion of studies from South/Southeast Asia outside of China decreased from 14% (17 studies) to 10% (13 studies).

For the 2009 to 2013 studies, the most common primary stated study purposes were to assess TDR prevalence (79%), to characterize sequences diversity for molecular epidemiologic purposes or vaccine development (14%), or to study transmission dynamics using sequence networks (3%). For the 2014 to 2019 periods, the proportion of studies designed for assessing TDR prevalence decreased to 45%, whereas studies to characterize sequence diversity for molecular epidemiologic purposes or vaccine development (32%) and to study transmission dynamics using sequence networks (16.1%) increased.

For the 2009 to 2013 studies, the most common recruitment sites were HIV clinics (55%), sentinel sites for HIV‐1 surveillance including blood donation centres, antenatal clinics, voluntary counselling testing sites and injection drug user clinics (33%) and regional reference or public health laboratories (10%). For the 2014 to 2019 studies, the most common recruitment sites were HIV clinics (41%), regional reference or public health laboratories (26%) and sentinel sites for HIV surveillance (22%). Fourteen (11%) of 2009 to 2013 and 22 (18%) of 2014 to 2019 studies consisted entirely of individuals with recent HIV‐1 infection.

During both study periods, approximately 92% of specimens was plasma, whereas the remaining specimens’ types were PBMCs and dried blood spots. Between 2014 and 2019, GenBank contained consensus NGS sequences from four studies with 50 or more ARV‐naïve persons. However, only one of these studies was included in this review because the consensus sequences used a mutation detection threshold that was below 1% in two studies and that was not reported in a third study. The consensus sequence from the fourth study used a 20% mutation‐detection threshold. There was a large NGS study in the NCBI Sequence Read Archive that did not have a consensus sequence in GenBank and was therefore not included in this analysis [16].

### 3.2 TDR prevalence

**Figure 3:**
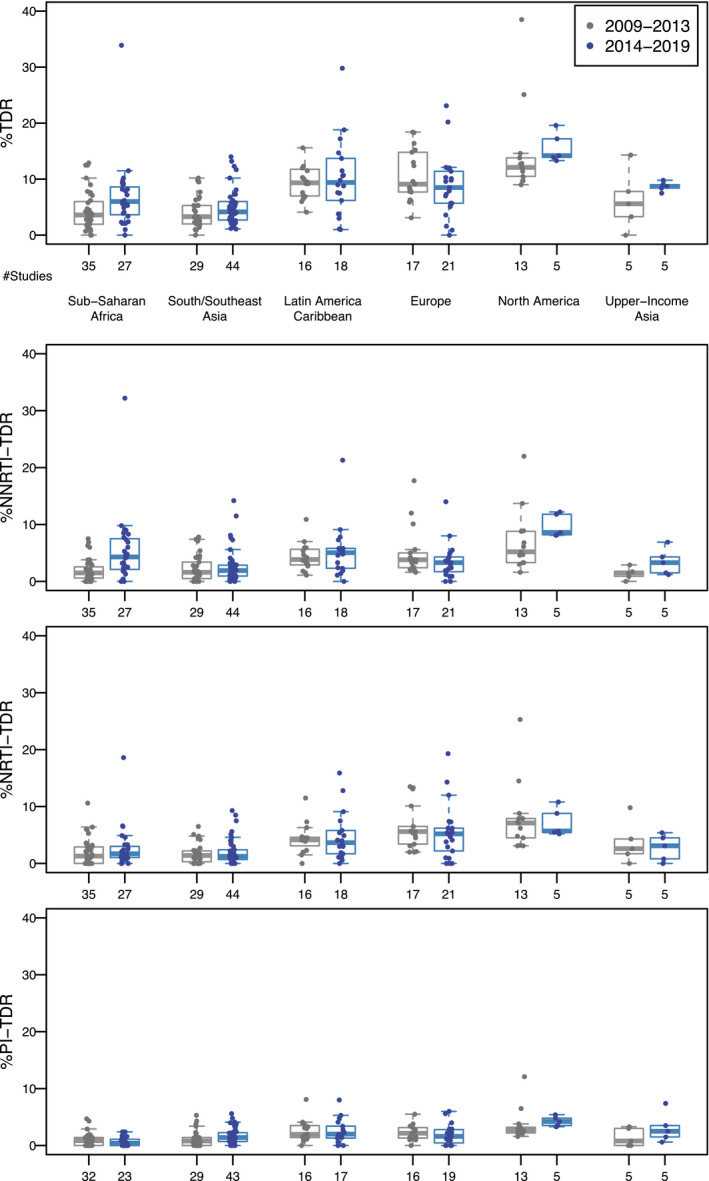
TDR prevalence estimates in studies containing ≥50 ART‐naïve persons published 2009 to 2013 and 2014 to 2019 for those regions with ≥5 studies.

In the 2014 to 2019 studies, the median study‐level TDR prevalence was 6.0% in sub‐Saharan Africa and 4.1% in South/Southeast Asia. By comparison, the median study‐level TDR prevalence in Europe, the upper‐income Asian countries, Latin America/Caribbean and North America, ranged from 8.5% to 14.2%. Between the two study periods, there was a statistically significant study‐level increase in overall TDR in sub‐Saharan Africa and North America, NNRTI TDR in sub‐Saharan Africa and PI TDR in South/Southeast Asia and North America, two‐class NRTI/NNRTI resistance in sub‐Saharan Africa (Table 2, Figure 3). There were no consistent regional or temporal differences in TDR prevalence estimates between different categories of recruitment site. There were no consistent regional or temporal differences in TDR prevalence between studies performed specifically for estimating TDR prevalence compared to those performed for other purposes.

**Table 2:** Comparisons of overall, NNRTI, NRTI and PI‐associated TDR prevalence: studies published in 2009 to 2013 and 2014 to 2019

	2009 to 2013 (n = 122)	2014 to 2019 (n = 125)	p‐value^a^
Sub‐Saharan Africa			
# Studies	35	27	
Overall	**3.6 (1.95 to 6)**	**6.0 (3.65 to 8.6)**	**0.01**
NNRTI	**1.5 (0.6 to 2.55)**	**4.3 (2.1 to 7.5)**	**<0.001**
NRTI	1.3 (0 to 2.9)	1.7 (1.05 to 3)	0.1
PI	0.85 (0 to 1.4)	0.4 (0 to 1.1)	0.9
NNRTI + NRTI	**0 (0 to 0.71)**	**1.04 (0 to 2.07)**	**0.002**
South/Southeast Asia			
# Studies	29	44	
Overall	3.3 (2.0 to 5.3)	4.15 (2.75 to 5.9)	0.1
NNRTI	1.6 (0.5 to 3.4)	1.9 (0.98 to 2.82)	0.3
NRTI	1.4 (0.3 to 2.2)	1.25 (0.7 to 2.4)	0.3
PI	**0.8 (0 to 1.4)**	**1.4 (0.7 to 2.25)**	**0.03**
NNRTI + NRTI	0 (0 to 1.37)	0.26 (0 to 0.92)	0.2
Latin America/Caribbean			
# Studies	16	18	
Overall	9.35 (7.05 to 11.62)	9.4 (6.53 to 13.15)	0.5
NNRTI	3.85 (3 to 5.53)	5.05 (2.32 to 5.8)	0.3
NRTI	4.15 (3.5 to 4.55)	3.65 (1.75 to 5.7)	0.7
PI	1.95 (1.4 to 3.4)	2 (1.3 to 3.4)	0.6
NNRTI + NRTI	0.47 (0 to 1.26)	0.72 (0 to 1.14)	0.4
Europe			
# Studies	17	21	
Overall	9.1 (7.7 to 14.8)	8.5 (5.7 to 11.4)	0.9
NNRTI	3.8 (2.4 to 5)	3.3 (1.7 to 4.3)	0.9
NRTI	5.6 (3.4 to 6.5)	5.2 (2.2 to 6.2)	0.8
PI	2.15 (1.35 to 3.08)	1.6 (0.45 to 2.8)	0.8
NNRTI + NRTI	1.34 (0.61 to 1.85)	0.84 (0 to 1.17)	0.9
North America			
# Studies	13	5	
Overall	**12.1 (10.5 to 13.8)**	**14.2 (13.9 to 17.2)**	**0.04**
NNRTI	5.2 (3.3 to 8.8)	8.6 (8.2 to 11.8)	0.06
NRTI	7.1 (4.5 to 7.9)	5.7 (5.5 to 8.8)	0.4
PI	**2.7 (2.2 to 3.2)**	**4.2 (3.5 to 4.8)**	**0.02**
NNRTI + NRTI	0.9 (0.73 to 2.35)	2.03 (1.9 to 2.15)	0.1
Upper‐Income Asian Countries			
# Studies	5	5	
Overall	5.6 (3.3 to 7.8)	8.7 (8.4 to 9)	0.1
NNRTI	1.5 (0.9 to 1.7)	3.3 (1.5 to 4.3)	0.09
NRTI	2.6 (1.7 to 4.3)	3.1 (0.8 to 4.5)	0.5
PI	0.8 (0 to 3)	2.5 (1.5 to 3.5)	0.1
NNRTI + NRTI	0 (0 to 0.26)	0.09 (0 to 0.19)	0.4

Data are median (IQR) of study‐level prevalence (%) individuals with any (overall), NNRTI‐, NRTI‐, PI‐ and two‐class NNRTI + NRTI‐associated SDRMs in studies published in 2009 to 2013 and 2014 to 2019 by region; Six studies from former Soviet Union countries, four studies from Middle East countries and two studies from Australia are not included in this table.

^a^ Median prevalence was compared using the Wilcoxon Rank Sum test; the significant increase with *p* < 0.05 is indicated in bold.

Figure 3 shows that there are six studies for which the overall TDR prevalence was outliers having overall TDR prevalence above 20%. Two of these were published in the United States between 2009 and 2013 [17, 18]. One study included samples from 91 chronically infected persons and the other included samples from 662 recently diagnosed persons. Four of the outlier studies were published between 2014 and 2019 including studies of 59 fisherman along Lake Victoria in Kenya, 141 persons prior to starting ART in Cuba, 118 newly diagnosed persons in Croatia and 289 hospitalized patients in Portugal [19, 20, 21, 22]. The study from Croatia contained a large cluster of persons with viruses containing a single SDRM T215S which was responsible for most of the resistant viruses in this study. The samples from the study in Portugal and from one of the U.S. studies contained a high proportion of persons with multiple SDRMs raising the question of whether some of the persons in these studies did not disclose previous ART use.

The individual patient‐level analysis of sequences obtained since 2009 included sequences from 39,663 individuals including sequences from 7338 individuals in the 2009 to 2013 studies and 32,325 individuals in the 2014 to 2019 studies. Table 3 bisects the pooled sequences into approximately equal numbers spanning the years 2009 to 2011 and 2012 to 2018 and compares the proportions of individuals with TDR during these time periods by region and ARV drug class. This analysis shows that the prevalence of overall TDR increased in sub‐Saharan Africa and North America but decreased in Europe. The prevalence of NNRTI TDR increased in sub‐Saharan Africa, North America and Latin America/Caribbean, whereas the prevalence of NRTI TDR decreased in Europe. The prevalence of two‐class NRTI/NNRTI resistance increased in sub‐Saharan Africa. The same significant trends were observed in the regression analysis relating sample year to TDR prevalence (Table 4).

**Table 3:** TDR in samples obtained since 2009: comparison of those obtained 2009 to 2011 and 2012 to 2018

	2009 to 2011 (n = 19,440)	2012 to 2018 (n = 20,223)	*p*‐value^a^
Sub‐Saharan Africa			
# Individuals	2909	3138	
Overall	**5.09**	**8.19**	**<0.001**
NNRTI	**3.61**	**7.3**	**<0.001**
NRTI	1.79	1.98	0.6
PI	0.68	0.42	0.2
NNRTI + NRTI	**0.83**	**1.43**	**0.03**
South/Southeast Asia			
# Individuals	7662	6320	
Overall	3.88	4.18	0.4
NNRTI	1.75	1.66	0.7
NRTI	1.55	1.38	0.4
PI	1.47	1.78	0.2
NNRTI + NRTI	0.67	0.47	0.1
Latin America/Caribbean			
# Individuals	1833	2799	
Overall	9.22	10.9	0.07
NNRTI	**4.64**	**6.25**	**0.02**
NRTI	4.15	4.32	0.8
PI	2.14	2.2	0.9
NNRTI + NRTI	1.31	1.18	0.7
Europe			
# Individuals	2776	3011	
Overall	9.94	7.9	0.007
NNRTI	3.67	2.99	0.2
NRTI	5.51	4.02	0.008
PI	2.52	2.31	0.7
NNRTI + NRTI	1.08	0.8	0.3
North America			
# Individuals	1773	1617	
Overall	**12.58**	**17.75**	**<0.001**
NNRTI	**7.11**	**11.13**	**<0.001**
NRTI	5.08	5.63	0.5
PI	**2.43**	**3.9**	**0.02**
NNRTI + NRTI	1.18	1.18	1
Upper‐Income Asian Countries			
# Individuals	1957	2800	
Overall	9.5	8.43	0.2
NNRTI	2.04	1.54	0.2
NRTI	4.39	4.79	0.6
PI	3.54	2.56	0.05
NNRTI + NRTI	0.1	0.18	0.7

Data are percent individuals with any (overall), NNRTI‐, NRTI‐, PI‐ and two‐class NNRTI + NRTI‐associated SDRMs in 2009 to 2011 and 2012 to 2018 by region; TDR prevalence in samples obtained from individuals in Middle East countries (n = 608 individuals), former Soviet Union countries (n = 374 individuals) and Australia (n = 86 individuals) are not included in this table.

^a^ The prevalence of overall, NRTI, NNRTI‐TDR in the samples obtained in 2012 to 2018 was compared with those in the samples obtained in 2009 to 2011 using Fisher’s Exact test; the significant increase with *p* < 0.05 is indicated in bold.

**Table 4:** Odds of TDR according to sample year: 2009 to 2018

Region^a^	Drug class	OR^b^ (95% CI)	*p*‐value
Sub‐Saharan Africa (n = 6047)	Overall	**1.12 (1.05 to 1.21)**	**0.002**
	NNRTI	**1.16 (1.08 to 1.26)**	**<0.001**
	NRTI	**1.12 (1.12 to 1.12)**	**<0.001**
	PI	0.87 (0.73 to 1.03)	0.1
South/Southeast Asia (n = 13,982)	Overall	1.01 (0.95 to 1.08)	0.7
	NNRTI	0.97 (0.88 to 1.07)	0.6
	NRTI	0.98 (0.89 to 1.08)	0.6
	PI	1.06 (0.97 to 1.15)	0.2
Latin America/Caribbean (n = 4632)	Overall	1.08 (0.99 to 1.18)	0.1
	NNRTI	**1.14 (1.03 to 1.27)**	**0.01**
	NRTI	1.06 (.095 to 1.18)	0.3
	PI	1.01 (0.94 to 1.09)	0.7
Europe (n = 5787)	Overall	0.95 (0.89 to 1.01)	0.07
	NNRTI	1.02 (0.93 to 1.13)	0.7
	NRTI	0.89 (0.83 to 0.97)	0.004
	PI	0.99 (0.89 to 1.10)	0.9
North America (n = 3390)	Overall	**1.10 (1.05 to 1.15)**	**<0.001**
	NNRTI	**1.10 (1.04 to 1.17)**	**<0.001**
	NRTI	**1.09 (1.01 to 1.18)**	**0.03**
	PI	**1.10 (1.02 to 1.19)**	**0.02**
Upper‐Income Asian Countries (n = 4757)	Overall	0.99 (0.94 to 1.04)	0.6
	NNRTI	0.93 (0.77 to 1.12)	0.4
	NRTI	1.02 (0.89 to 1.17)	0.7
	PI	0.92 (0.85 to 1.00)	0.06

^a^ The number of individuals is indicated in each region (n)

^b^ for each region, a generalized linear mixed model was used to assess the yearly change in the odds (OR) of TDR accounting for study heterogeneity using the R package lme4. The model included the categorical outcome variable indicating the presence or absence of TDR and the two explanatory variables, the sample year as a fixed‐effect term and the study as a random‐effect term; The significant increase with *p* < 0.05 is indicated in bold.

### 3.3 Distribution of DRMs

Eight NNRTI‐associated mutations had a prevalence above 0.1%. The prevalence of three of these mutations (K103N, Y181C and Y188L) increased by more than 2‐fold in sub‐Saharan Africa (Table 5). Among the NRTI resistance mutations, K65R had a significant increase in prevalence from 0.03% to 0.35% in sub‐Saharan Africa, whereas M184V had a non‐significant increase from 0.65% to 1.05%. The NNRTI resistance mutations K103N and V106M also increased in prevalence in the Latin America/Caribbean region. Of note, K65R and V106M are preferentially selected in subtype C viruses which are highly prevalent in sub‐Saharan Africa [23, 24]. There were no significant increases of any DRMs in South/Southeast Asia. The Table S3 lists the prevalence of each DRM in each region by time period.

**Table 5:** Temporal trends in prevalence of individual DRMs and DRM patterns in three low‐ and middle‐income country regions

DRM	Sub‐Saharan Africa		South/Southeast Asia		Latin America/ Caribbean	
	2009 to 2011 (n = 2909)	2012 to 2018 (n = 3138)	2009 to 2011 (n = 7662)	2012 to 2018 (n = 6320)	2009 to 2011 (n = 1833)	2012 to 2018 (n = 2799)
NNRTIs						
K103N	**2.06**	**5**	0.67	0.59	**2.45**	**4.29**
V106M	**0.03**	**0.8**	0.1	0.17	0.05	0.14
Y181C	0.52	0.67	0.59	0.35	0.38	0.39
Y188L	**0.07**	**0.22**	0.1	0.06	0.22	0.18
G190A	0.45	0.76	0.26	0.32	0.76	1.11
Other SDRMs	0.79	1.08	0.38	0.46	1.47	1.46
NRTIs						
K65R	**0.03**	**0.35**	0.05	0.09	0	0.04
M184V	0.65	1.05	0.48	0.43	0.76	0.57
T215Y/F	0.21	0.16	0.17	0.13	0.22	0.14
Any TDF	0.34	0.73	0.64	0.49	0.65	0.54
New TDF	0.14	0.32	0.39	0.22	0.44	0.39
Any TAMs	1.07	0.8	0.97	0.82	3.16	3.11
PIs						
M46IL	0.2	0.07	0.99	1.09	0.66	0.9
V82A	0	0	0.07	0.02	0.05	0.26
L90M	0.04	0.03	0.14	0.05	0.71	0.26

Data are percent individuals with DRMs and DRM patterns in 2009 to 2011 and 2012 to 2018 in three low‐ and middle‐income country regions, sub‐Saharan Africa, South/Southeast Asia and Latin America/Caribbean. Underline: ≥2‐fold increased prevalence. Bold: *p* < 0.05.

## 4 Discussion

During the six‐year period between 2014 and 2019, 125 studies comprising 50 or more ARV‐naïve persons were submitted to GenBank. The populations and TDR prevalence in these studies were compared to data from 122 studies meeting the same criteria submitted to GenBank between 2009 and 2013. In sub‐Saharan Africa, there was an increase in the median study‐level prevalence of overall and NNRTI‐associated TDR in the 2014 to 2019 period, whereas individual patient‐level analyses demonstrated these increases and an increase in dual NRTI/NNRTI‐associated TDR. Additional significant trends included an increase in NNRTI‐associated TDR in the Latin America/Caribbean region and an increase in overall, NNRTI and PI‐associated resistance in North America by both study‐level and individual patient‐level analysis.

During both time periods, there was a gradient in the prevalence of TDR with the lowest levels in South/Southeast Asia and sub‐Saharan Africa; intermediate levels in Europe, the Latin America/Caribbean region and the upper‐income Asian countries; and the highest levels in North America. Between the two time periods, TDR prevalence levels in sub‐Saharan Africa increased relative to South/Southeast Asia, whereas those in Europe decreased relative to the Latin America/Caribbean region.

The high rates of TDR in North America reported here are consistent with recent U.S. CDC surveys presented at scientific meetings but not included in our analysis [25, 26]. The reasons for the higher TDR rates in the United States than in other upper‐income countries may be due to the higher retention in care outside of the United States, where ART is provided free of charge [27, 28]. Although PrEP has been increasingly used in the United States, its use was unlikely to have influenced TDR incidence because the mutations selected by PrEP, M184V and K65R, occurred rarely throughout the study [29]. TDR is a less significant public health problem in upper‐income countries because pretherapy genotypic resistance testing is usually available to identify persons with TDR and to adjust first‐line therapy accordingly. Moreover, TDR in upper‐income countries often results from the onward transmission of strains containing mutations associated with ARVs that have been used in many years [26, 30, 31, 32, 33]. In LMICs, however, TDR strains are more likely to contain mutations derived directly from treated persons such M184V and K65R, that have not had the opportunity to revert to wild‐type [34].

This study also reveals trends in how sequence data on HIV‐1‐associated TDR are being obtained. First, the proportion of studies from sub‐Saharan Africa decreased from 29% to 22% and those from South/Southeast Asia other than China decreased from 14% to 10%. Second, there were several differences in study design that influenced the nature of the population studied during the two time periods. Compared with the earlier study period, a smaller proportion of studies published between 2014 and 2019 were performed expressly for the purpose of monitoring TDR (79% vs. 45%; Fisher Exact test; *p* < 0.001) and fewer were performed at sentinel sites for HIV‐1 surveillance (33% vs. 22%; Fisher Exact test; *p* = 0.09). Although there were no consistent regional or temporal differences in TDR prevalence between studies performed for assessing TDR prevalence and those performed for other purposes, the above trends suggest that there is a reduced investment in studies designed specifically for TDR surveillance particularly in those regions for which TDR prevalence data are most needed.

In contrast to the most recent WHO systematic review [6], we excluded studies containing ART‐experienced persons presenting for initial therapy. During the 2014 to 2019 study period, nine such studies were excluded – seven from sub‐Saharan Africa and two from South/Southeast Asia. Although 7% to 40% of persons in these studies had a history of previous ARV use, the median PDR prevalence in these studies (6.4%; range: 3.4% to 13.3%) did not differ from the TDR prevalence in the 125 studies containing entirely ART‐naïve populations. However, PDR prevalences can be several‐fold higher among persons with previous ARV compared to those documented to be ART‐naïve [9, 35] because they depend in part on the extent to which patients cycle in and out of care rather than the likelihood that a person will be primarily infected by a drug‐resistant virus. Therefore, for epidemiological purposes it remains valuable to monitor drug resistance in ART‐naïve individuals to document the prevalence and patterns of TDR strains, assess “hot spots” of transmission of drug‐resistant virus to identify individuals at risk for having TDR at time of diagnosis and to implement public health measure to interrupt TDR transmission.

Of the 247 studies in our review, 132 (53.4%) were included in the 2018 WHO meta‐analysis [6]. The additional 115 studies in our review included 37 studies from LMICs published since 2016 and 78 studies from upper‐income countries. The WHO meta‐analysis also contained 190 studies that we excluded because they were published prior to 2009 or were unpublished, contained fewer than 50 persons, lacked publicly available sequences, and/or included persons with previous ARV use.

Our approach to analysing TDR trends has several limitations. First, some countries are better able to conduct surveillance studies. This points to the importance of the WHO global surveillance programme which supports surveillance studies in resource‐limited areas [36]. However, the number of studies conducted by WHO supported laboratories is not high [9]. Second, by relying entirely on publicly available sequences, we were confined to the subset of studies submitted to GenBank. This can introduce a bias in that researchers from some regions may be more likely to submit their sequences to GenBank. For example a large proportion of the studies from South/Southeast Asia was from China. Third, not all studies were performed in order to determine TDR prevalence. Although TDR prevalence estimates did not differ between those conducted for surveillance purposes and those conducted for other reasons, differences in study design may explain some of the heterogeneity within each of the regions. Additionally, non‐disclosed past ART use may have influenced TDR estimates in our studies as well as in PDR surveys.

The availability of sequences from each study participant made it possible to track geographic and temporal trends in the prevalence of SDRMs as well as mutations that are not SDRMs such as the recently identified but as yet uncommon non‐polymorphic tenofovir‐associated mutations [15]. The availability of sequences from the studies presented here make it possible for other researchers to use these data to analyse the association of different mutations with different subtypes, the genetic relatedness of sequences containing different DRMs and the proportions of positions in each sequence containing ambiguities – an indicator of recent infection [10]. As more research and clinical laboratories use NGS for genotypic resistance testing, it will be important for their data to be submitted to the NCBI Sequence Read Archive so that their data can be analysed using different mutation thresholds. Consensus NGS sequences submitted to GenBank may be difficult to be included in meta‐analyses, if their mutation detection thresholds are set too low.

## 5 Conclusions

Published studies of HIV‐1 pol sequences in ART‐naïve populations provide important insights into global TDR trends. However, the decreasing proportion of studies from the hardest hit low‐ and middle‐income country regions and the shift away from sentinel sites for recent infection suggests that global TDR surveillance efforts require renewed emphasis.

## Competing Interests

We declare no competing interests.

## Authors’ Contributions

SYR and RWS designed the study, analysed the data, and were the primary writers of the paper. SYR, SGK, GB and JCS screened titles, abstracts and full‐text articles for inclusion and extracted data from included studies. SYR performed statistical analysis. SGK, GB and MRJ provided oversight, critical feedback and interpretation of results. All authors contributed to writing the manuscript and approved the final version.

## Supporting information


**Table S1.** Search terms and strategy

**Table S2.** Studies which met inclusion criteria

**Table S3.** Prevalence of each DRM in each region by time period
