# Supplementary material for: HIV‐1 transmitted drug resistance surveillance: shifting trends in study design and prevalence estimates
Source: J Int AIDS Soc. 2020 Sep 16;23(9):e25611. doi: 10.1002/jia2.25611 (PMC7507012; doi:10.1002/jia2.25611)
Supplement: Supplementary file 1 — Table S1. Search terms and strategy [file JIA2-23-e25611-s001.docx]

**Table S1: Search terms and strategy^†^**

| **GENBANK** | |
| --- | --- |
| HIV-1 group M RT sequences | Step 1: Downloaded GenBank-format flat files of viral sequences  Step 2: Conversion to BLAST searchable database using MAKEBLASTDB (in NCBI blast+ package)  Step 3: TBLASTN (in NCBI blast+ package) search using HIV-1 group M subtype B RT consensus amino acid sequence  Step 4: Sequences containing RT +/- protease and accompanying annotations retrieved  Step 5: Search results grouped into submission sets containing the same “Title” and “Authors” |
| **^†^**The same procedure was used and described in the publications below:  Rhee S-Y, Shafer RW. Geographically-stratified HIV-1 group M pol subtype and circulating recombinant form sequences. Sci. Data5:180148 doi: 10.1038/sdata.2018.148(2018)  Rhee, S-Y, Margeridon-Thermet S, Nguyen MH, Liu TF, Kagan RM, Beggle B, et al, Hepatitis B virus reverse transcriptase sequence variant database for sequence analysis and mutation discovery. Antiviral Res. (2010), doi:10.1016/j.antiviral.2010.09.012 | |
